# Recurrent Horner’s Syndrome and Cranial Nerve Involvement Following Epidural Analgesia During Labor: A Case Report

**DOI:** 10.1155/cria/4477329

**Published:** 2026-11-02

**Authors:** Cristine Cavegn, Evgenia Anastasiadou, Maria Lünnemann, Romana Brun, Daniel Anthony Button, Caveh Madjdpour

**Affiliations:** ^1^ Department of Anesthesiology Kantonsspital Winterthur Brauerstrasse 15 Winterthur 8400 Switzerland ksw.ch; ^2^ Department of Obstetrics and Gynecology Kantonsspital Winterthur Brauerstrasse 15 Winterthur 8400 Switzerland ksw.ch

**Keywords:** case report, Horner’s syndrome, obstetric anesthesia, pregnancy-associated stroke

## Abstract

We report a rare case of a 33‐year‐old primigravida developing an extensive cephalad spread of epidural local anesthetic resulting in bilateral Horner’s syndrome and cranial nerve involvement during labor and cesarean delivery. Urgent neuroimaging excluded intracranial hemorrhage. Due to persistent focal neurological symptoms during second‐stage labor and inability to exclude a life‐threatening cerebrovascular pathology, cesarean delivery was performed for maternal indication. Following epidural top‐up for surgical anesthesia, symptoms recurred and progressed bilaterally, thereby supporting extensive cephalad spread as the underlying mechanism. Postpartum MRI and MR angiography revealed no cerebrovascular abnormalities. This case illustrates the diagnostic challenge of acute neurological symptoms during labor, where pregnancy‐associated stroke risk necessitates exclusion of life‐threatening pathology despite time constraints. Recognition of pregnancy‐related factors predisposing to extensive epidural spread, combined with interdisciplinary collaboration and appropriate neuroimaging, enabled safe management.

## 1. Introduction

Acute neurological symptoms arising during labor are uncommon but represent a time‐critical situation in which obstetric, anesthetic, and neurologic priorities must be balanced to protect maternal and fetal safety [1]. Labor epidural analgesia is widely used; however, transient neurological signs, including Horner’s syndrome, may occasionally occur following neuraxial techniques [2]. In the obstetric setting, the presence of Horner’s syndrome necessitates the exclusion of potentially serious central neurological causes [3]. The physiological changes of pregnancy, including hypercoagulability and altered hemodynamics, increase the risk of cerebrovascular events such as ischemic stroke and venous thrombosis during the peripartum period, which may present with acute focal neurological deficits [4]. When such symptoms develop intrapartum, clinical decision‐making is often challenged by the need to act before a definitive neurological diagnosis can be established. Diagnostic pathways during labor are often constrained by time pressure, maternal discomfort, and the progression of labor, particularly when advanced cervical dilation limits the possibility of prolonged evaluation. Under these conditions, obstetric management must prioritize maternal safety despite persistent diagnostic uncertainty. From an obstetric perspective, the second stage of labor involves repeated maternal pushing efforts that may influence intrathoracic pressure and cerebral hemodynamics [5]. When acute intracranial pathologies cannot be excluded, the potential impact of sustained Valsalva maneuvers becomes a relevant consideration in delivery planning. Under such conditions, decisions regarding the mode of delivery must be guided by individualized risk assessment rather than predefined algorithms.

## 2. Case Presentation

A 33‐year‐old primigravida parturient at 40 + 2 weeks of gestation presented in spontaneous labor. Her pregnancy had been uneventful. Past medical history was notable only for a bicycle accident at 20 + 5 weeks of gestation resulting in a right‐sided radial fracture, after which obstetric follow‐up remained unremarkable. She had no chronic medical conditions and was not taking regular medication. At cervical dilation of 4 cm, labor epidural analgesia was requested for labor pain. Standard monitoring was applied, and the procedure was performed in the sitting position at the L3‐L4 interspace using the loss‐of‐resistance‐to‐saline technique. The epidural space was identified at a skin‐to‐epidural‐space depth of 5 cm, and the catheter was subsequently threaded 5 cm into the epidural space. Epidural catheter placement was uneventful. There was no cerebrospinal fluid or blood aspiration. No test dose was administered prior to initiation of analgesia. Initial analgesia was established with ropivacaine 1 mg/mL and fentanyl 2 μg/mL (20 mL), followed by patient‐controlled epidural analgesia (PCEA) with a 15‐min lockout interval and programmed intermittent epidural bolus (PIEB) of 5 mL every 45 min, providing adequate pain relief without immediate complications. The first stage of labor progressed appropriately, and fetal monitoring remained physiological.

Approximately 1 h after catheter placement and initiation of labor epidural analgesia with cumulative PCEA/PIEB volume of up to 20 mL, the patient reported new‐onset symptoms consisting of a sensation of swelling of the left eyelid and sensory impairment of the left upper limb. Neurological examination, with sensory assessment performed using cold sensation and light touch, revealed a symmetrical sensory blockade extending from thoracic level T4 to lumbar level L5. Motor assessment demonstrated mild weakness (Medical Research Council grade M4) of the left triceps and intrinsic hand muscles. Notably, signs consistent with cranial nerve involvement were observed, with prominent findings including reduced sensation in the distribution of the left trigeminal nerve and a left‐sided Horner’s syndrome characterized by miosis, ptosis, and conjunctival injection.

A multidisciplinary evaluation involving obstetrics, neurology, and anesthesiology was initiated immediately. Given the acute onset of focal neurological deficits, an urgent noncontrast cranial computed tomography (CT) scan was performed when cervical dilation had reached 9 cm. Although no intracranial hemorrhage was detected, other acute cerebrovascular conditions, including ischemic stroke, cerebral venous thrombosis, and arterial dissection, could not be reliably excluded. Fetal monitoring remained reassuring. As labor progressed toward the second stage, the obstetric and anesthetic teams were concerned about the potential effects of sustained maternal pushing in the presence of an unidentified neurological condition. Given the need for timely decision‐making and the possibility that further neurological investigation or treatment might become necessary, an interdisciplinary decision was made to proceed with secondary cesarean delivery for maternal indication. Before topping up the labor epidural anesthesia, the patient reported near‐complete resolution of her previous symptoms. Objective reassessment confirmed full motor strength (M5) in both upper extremities, symmetrical facial sensation, and only subtle residual left‐sided Horner’s syndrome.

The decision to extend the existing epidural block for cesarean anesthesia was made following interdisciplinary risk–benefit assessment. At the time of this decision, the neurological symptoms had almost completely resolved. The epidural catheter had provided effective analgesia with a bilateral sensory block, and maternal hemodynamics had remained stable throughout the initial episode. Although the underlying cause of the neurological findings remained uncertain, the initial cranial CT had revealed no evidence of intracranial hemorrhage. Epidural extension was considered a reasonable alternative to general anesthesia, particularly because it would preserve consciousness and allow continued clinical neurological assessment. Given the possibility of recurrent neurological symptoms or unexpectedly extensive neuraxial blockade, the procedure was undertaken with readiness to convert to general anesthesia if clinically indicated.

For surgical anesthesia, the epidural catheter was topped up with lidocaine 20 mg/mL (cumulative dose 300 mg) combined with adrenaline 5 μg/mL and a cumulative dose of 100 μg fentanyl was administered epidurally. Following administration, the neurological symptoms reappeared and progressed. Initially beginning on the left side, the signs subsequently extended contralaterally. The patient reported heaviness of the eyelids and recurrent facial numbness, as well as subjective difficulty speaking. Clinical examination again demonstrated Horner’s syndrome, facial flushing, conjunctival injection, and reduced facial sensation. The progression from unilateral to bilateral symptoms suggested cephalad spread of local anesthetic with involvement of upper thoracic and possibly cervical sympathetic fibers. Hemodynamic parameters remained stable throughout the episode, and oxygen saturation was preserved. No evidence of systemic local anesthetic toxicity (e.g., tinnitus, metallic taste, seizures, and arrhythmias) was observed. A repeat vaginal examination performed after the epidural top‐up and immediately before surgery confirmed full cervical dilation (10 cm), with the fetal head at station −1 in the occiput anterior position. Despite complete cervical dilation, operative vaginal delivery was not considered feasible because of the high fetal head station. The cesarean delivery proceeded without further complications.

Postoperatively, after discontinuation of epidural local anesthetic administration, all neurological symptoms resolved completely without neurological sequelae. Neurology consultation recommended magnetic resonance imaging (MRI) of the brain with angiographic sequences to exclude a cerebrovascular etiology. MRI of the brain, face, neck, and MR angiography were subsequently performed during the postpartum course and demonstrated an age‐appropriate normal appearance of the neurocranium, with no evidence of acute ischemia, vascular dissection, cerebral venous thrombosis, vasogenic edema, or intracranial hemorrhage. The postoperative course was uncomplicated.

## 3. Discussion

This case illustrates extensive cephalad spread of epidural local anesthetic causing bilateral Horner’s syndrome accompanied by cranial nerve palsies, a rare but clinically important presentation. While isolated epidural‐associated Horner’s syndrome is a recognized complication of neuraxial anesthesia, concomitant cranial nerve involvement substantially broadens the differential diagnosis and necessitates urgent interdisciplinary evaluation to exclude central neurological pathology, particularly cerebrovascular events [6]. The complete and reproducible resolution of symptoms following regression of the neuraxial block ultimately supported extensive epidural spread as the underlying mechanism and distinguished it from structural neurological disease [3, 7].

Several pregnancy‐related physiological and anatomical changes may predispose parturients to extensive cephalad spread of epidurally administered local anesthetics. Epidural venous engorgement and increased tissue volume may reduce the available epidural space, potentially facilitating the longitudinal spread of local anesthetic. In addition, transient increases in epidural pressure associated with uterine contractions may further influence drug distribution. Hormonal changes during pregnancy, particularly elevated progesterone concentrations, have also been proposed to increase neuronal sensitivity to local anesthetics, potentially contributing to a more extensive neuraxial block. Anatomical variability may provide a further explanation for the clinical presentation. Transverse and lateral epidural septa may influence the distribution of local anesthetic, while individual differences in the origin and course of the oculosympathetic pathways may contribute to the variable presentation of Horner’s syndrome [6]. In particular, sympathetic pathways supplying the eye may originate from spinal cord levels as low as T4, potentially explaining the occurrence of ocular sympathetic blockade following a lumbar epidural injection [8]. Furthermore, autonomic blockade may extend several dermatomes above the sensory level, meaning that cephalad spread of local anesthetic sufficient to produce a sensory block to T4, as assessed by the absence of cold sensation, could have reached the preganglionic sympathetic fibers of the oculosympathetic pathway, resulting in its disruption [9]. Differences in sympathetic innervation between the two sides may also contribute to asymmetric initial manifestations [6]. In the present case, the initial unilateral symptoms, subsequent bilateral progression following epidural top‐up, and complete resolution after cessation of epidural local anesthetic administration are consistent with extensive cephalad spread. A possible neuroanatomical explanation of trigeminal nerve involvement is that the spinal trigeminal nucleus and tract are not contained within the brainstem only but extend caudally into the upper cervical spinal cord as far down as the C4 spinal cord segments [10]. Therefore, trigeminal nerve impairment might be caused by local anesthetic reaching the upper cervical segments of the spinal cord without it spreading up to the brainstem. A spread up to C4 would be compatible with the left upper extremity weakness observed in our patient. Nevertheless, the proposed pregnancy‐related and anatomical factors remain plausible explanatory mechanisms rather than directly demonstrated causes in this patient.

Several alternative diagnoses were considered during the initial neurological assessment. Acute ischemic stroke could not be excluded by noncontrast CT alone, although subsequent MRI showed no evidence of acute ischemia [11]. Intracranial hemorrhage was ruled out by the initial CT examination. Cerebral venous sinus thrombosis was also considered, particularly given the hypercoagulable state of pregnancy. MRI with arterial and venous vascular assessment showed no evidence of cerebral venous sinus thrombosis or arterial dissection [4]. Cervical artery dissection, especially involving the internal carotid artery, was another important consideration in view of the acute Horner’s syndrome, but MR angiography revealed no vascular dissection. Although high spinal anesthesia due to inadvertent intrathecal placement or subsequent catheter migration was briefly considered, it was deemed highly unlikely given the absence of lower extremity motor blockade, stable hemodynamics and lack of respiratory compromise. Local anesthetic systemic toxicity was considered less likely given the absence of characteristic neurological prodromes, seizures, arrhythmias, or cardiovascular instability. Overall, the reproducible association between epidural dosing and symptom recurrence favored extensive cephalad spread of local anesthetic.

Pregnancy‐associated stroke, although uncommon (30 per 100,000 pregnancies), remains an important cause of maternal morbidity and mortality [11, 12]. In this case, the main obstetric concern was the possibility of an underlying cerebrovascular condition that could not be reliably excluded by noncontrast CT. The anticipated second stage of labor raised concerns regarding the effects of repeated Valsalva maneuvers on cerebral hemodynamics [5]. This concern arose from the assessment of the obstetric and anesthetic teams rather than from an explicit neurological recommendation to avoid maternal pushing. At the time of the initial decision, cervical dilation was 9 cm and fetal monitoring remained reassuring. Although the neurological symptoms had substantially improved before surgical anesthesia, their underlying cause remained uncertain. This improvement alone was not considered sufficient to exclude a cerebrovascular event or to reverse the initial decision regarding the mode of delivery. Although full dilation was subsequently confirmed immediately before surgery, the fetal head remained at station −1 in the occiput anterior position, making operative vaginal delivery unsuitable. Continuing toward spontaneous vaginal birth would have involved further fetal descent and potentially prolonged maternal pushing. The possibility that additional urgent neurological evaluation or treatment might become necessary was also considered. Cesarean delivery was therefore chosen as an individualized precautionary measure in the setting of persistent diagnostic uncertainty.

The decision to use the existing epidural catheter for surgical anesthesia warrants critical consideration. Although the temporal association between epidural administration and the initial neurological symptoms suggested a neuraxial mechanism, the presence of additional focal deficits precluded a definitive diagnosis at that stage. The bilateral sensory blockade, effective analgesia, stable hemodynamics, and substantial regression of neurological symptoms were considered reassuring findings. Nevertheless, these observations could not exclude an atypical distribution of local anesthetic, including possible subdural spread, or eliminate the risk of recurrent neurological symptoms following further epidural administration.

The choice between extending the existing epidural block or proceeding with general anesthesia therefore required an individualized assessment of the potential risks and benefits. Preserving consciousness allowed continued clinical neurological monitoring, whereas general anesthesia would have temporarily precluded such assessment. Conversely, further epidural dosing carried the risk of recurrent or more extensive neuraxial blockade. The recurrence and bilateral progression of neurological symptoms following the epidural top‐up subsequently strengthened the presumed association with neuraxial local anesthetic administration, although the precise anatomical mechanism remained unconfirmed. Importantly, this observation became available only after the anesthetic technique had been selected and therefore cannot retrospectively establish the safety or appropriateness of the initial decision. This case highlights the importance of individualized decision‐making, recognition of diagnostic uncertainty, and preparedness to modify the anesthetic strategy when unexpected neurological findings occur during labor.

This case demonstrates that the principal challenge is not recognizing epidural‐associated Horner’s syndrome but managing Horner’s syndrome accompanied by cranial nerve involvement while potentially serious neurological pathology remains unexcluded. Awareness of pregnancy‐related factors predisposing to extensive epidural spread, together with prompt neuroimaging, close interdisciplinary collaboration, and ongoing reassessment, enabled safe maternal and neonatal outcomes.

## Author Contributions

Cristine Cavegn: conceptualization, writing–original draft, and writing–review and& editing. Evgenia Anastasiadou: conceptualization, writing–original draft, and writing–review and editing. Maria Lünnemann: conceptualization and writing–review and editing. Romana Brun: validation and writing–review and editing. Daniel Anthony Button: validation and writing–review and editing. Caveh Madjdpour: conceptualization, supervision, validation, and writing–review and editing.

## Funding

No funding was received for this manuscript.

## Consent

This study was published with the written informed consent of the patient.

## Conflicts of Interest

The authors declare no conflicts of interest.

## Data Availability

Data sharing is not applicable to this article as no datasets were generated or analyzed during the current study.

## References

[1] Elgendy I. Y. , Bukhari S. , Barakat A. F. , Pepine C. J. , Lindley K. J. , and Miller E. C. , Maternal Stroke: a Call for Action, Circulation. (2021) 143, no. 7, 727–738, 10.1161/CIRCULATIONAHA.120.051460. PMC8049095 33587666

[2] Rabinovich A. , Abedelhady R. , Mazor M. , Piura B. , and Margolin E. , Horner’s Syndrome Following Epidural Analgesia During Labor: Report of Six Cases, European Journal of Obstetrics & Gynecology and Reproductive Biology. (2010) 149, no. 2, 229–230, 10.1016/j.ejogrb.2009.11.020. 20031295

[3] Turbelin C. and Mallat J. , Recurrent Horner′s syndrome Following Epidural Analgesia for Labor: a Case Report, Medicine. (2020) 99, no. 5, 10.1097/MD.0000000000018862. PMC7004573 32000389

[4] Saposnik G. , Bushnell C. , Coutinho J. M. et al., Diagnosis and Management of Cerebral Venous Thrombosis: a Scientific Statement from the American Heart Association, Stroke. (2024) 55, no. 3, e77–e90, 10.1161/STR.0000000000000456. 38284265

[5] Keating N. , Rooney G. , McGuinness S. , and McAuliffe F. M. , A Review of the Effects of Valsalva Manoeuvre in Labour and Birth, Obstetric Medicine. (2025) 18, no. 1, 4–8, 10.1177/1753495X241269121. PMC11563555 39553189

[6] Chambers D. J. and Bhatia K. , Horner′s syndrome Following Obstetric Neuraxial Blockade-a Systematic Review of the Literature, International Journal of Obstetric Anesthesia. (2018) 35, 75–87, 10.1016/j.ijoa.2018.03.005. 29657082

[7] Narouze S. N. , Basali A. , Mandel M. , and Tetzlaff J. E. , Horner′s syndrome and Trigeminal Nerve Palsy After Lumbar Epidural Analgesia for Labor and Delivery, Journal of Clinical Anesthesia. (2002) 14, no. 7, 532–534, 10.1016/s0952-8180(02)00406-3. 12477590

[8] Ray B. S. , Hinsey J. C. , and Geohegan W. A. , Observations on the Distribution of the Sympathetic Nerves to the Pupil and Upper Extremity as Determined by Stimulation of the Anterior Roots in Man, Annals of Surgery. (1943) 118, no. 4, 647–655, 10.1097/00000658-194310000-00013. PMC1617782 17858298

[9] Fink B. R. , Mechanisms of Differential Axial Blockade in Epidural and Subarachnoid Anesthesia, Anesthesiology. (1989) 70, 851–858. 10.1097/00000542-198905000-00023 2719320

[10] Ray B. S. and Wolff H. G. , Experimental Studies on Headache: Pain-Sensitive Structures of the Head and Their Significance in Headache, Archives of Surgery. (1940) 41, no. 4, 813–856, 10.1001/archsurg.1940.01210040002001.

[11] Miller E. C. , Bello N. A. , Chen P. R. et al., Prevention and Treatment of Maternal Stroke in Pregnancy and Postpartum: a Scientific Statement from the American Heart Association, Stroke. (2026) 57, no. 4, 10.1161/STR.0000000000000514. 41603019

[12] Mehta L. S. , Warnes C. A. , Bradley E. et al., Cardiovascular Considerations in Caring for Pregnant Patients: a Scientific Statement from the American Heart Association, Circulation. (2020) 141, no. 23, 10.1161/CIR.0000000000000772. 32362133

